# Address

**Published:** 1844-12

**Authors:** James Taylor


### ARTICLE XI.

*Address.* Delivered on Saturday evening, June 23d, 1844, before the Cincinnati Association of Dental Surgeons. By James Taylor, M. D., D. D. S.

Mr. President:

In my lecture this evening I purpose calling the attention of this society for a short time to a late work on dentistry, by Paul B. Goddard, M. D., demonstrator of anatomy in the University of Pennsylvania, lecturer on anatomy, &c., &c., aided in the practical part by Joseph E. Parker, dentist.

Every work treating on dental science should demand of us a careful and attentive perusal. It is only by a thorough pathological and physiological knowledge of our profession that we must expect properly to be guided in our manipulations, and without this knowledge we will often, not only injure our patients, but degrade ourselves and profession in the estimation of an enlightened and confiding public.

It is lamentable that the ground work of our profession has received so little attention from those who have spent their time, and I might say, lives, in its practice. I hope the time has passed when a few weeks spent in learning the technical names of the dental organism, and in collecting a case of forceps, turnkeys and pluggers, shall be considered sufficient to qualify even the "seventh son" for the duties and responsibilities of our profession.

I must confess that a somewhat laborious practice of sixteen years and upwards, (with at least a smattering knowledge of medicine to start on,) has not been sufficient to give me that success in practice, or that confidence in a "royal road to knowledge," I could desire.

Let us hope, however, Mr. President, that, with the aid of improved microscopes, the magnetic telegraph and phreno-mnemotechny, the "rising generation" may travel on this road, if not royal, at least electric, faster than we have done.

The structure of the teeth has of late years received considerable attention, not only in a pathological, but geological point of view. Their "peculiar conformation indicate so exactly the type of animal to which they belong, that they are found to furnish the best characteristic marks by which to classify the members of the animal kingdom."

The researches of Cuvier, Rousseau, Purkinje, Müller, professor Retzius, and, still more recent, those of Mr. Nasmyth, must be read with considerable interest by every dentist. Not that they have settled the question of the structure of the teeth, but they point out the road by which much important knowledge is to be acquired.

I look forward to the remaining researches of Mr. Nasmyth on this subject with considerable interest. The opportunities he possesses for investigation, and that commendable perseverance which mark his already published treatise, speak well for some forthcoming light on this department of science.

Mr. President, the objects of this address will not permit me to indulge in giving the views and theories of the several authors I have named. This would be a more pleasant task than the one I have assumed for the duties of this evening.

The theory of the tubular structure of the teeth, first taught, I believe, by Leuwenhock, after laying in the dust and rubbish of neglected and forgotten things for more than a century and a half, has lately, by the aid of powerful microscopes, been revived and pursued with a diligence promising much good to dental science.

The experiments of Dr. Goddard sustain those of several of the scientific authors I have named, but particularly those of Professor Retzius. There is, however, one defect, I think, in all the experiments that have fallen under my observation, and which, I think, if properly attended to, would go far to settle many of the discrepancies we notice in the different experiments made.

This defect is in not paying proper regard to the age of the individual from whose mouth the teeth experimented upon are taken. I believe that the teeth of the young are much more porous, and probably contain more cartilage than those of the aged.

Allow me here to present the analysis of Mr. Pepys, which is as follows:

| | Roots of the Teeth. | Teeth of Adults. | First Teeth of Children. |
|---|---|---|---|
| Phosphate of lime | 58 | 64 | 62 |
| Carbonate | 4 | 6 | 6 |
| Cartilage | 28 | 20 | 20 |
| Loss | 10 | 10 | 12 |

Here we see that the roots of the teeth contain six parts less of the phosphate of lime than the teeth of adults, four parts less than the first teeth of children, and eight parts more of cartilage than either. It would appear from this that the teeth of children, which are only to answer a temporary purpose, have not as much of the animal matter which enters into bone as the roots of the teeth.

Might we not, by a careful analysis of the teeth from the mouth of an individual who has just received his permanent ones, obtain the thirty-three per cent. of animal matter, which the doctor thinks necessary to constitute bone?

We know that the dental cavity becomes almost entirely obliterated at an advanced age, and that there is a corresponding less degree of sensibility in the teeth as we approach the decline of life.

These remarks are made, Mr. President, merely introductory, as it were, to the all-important doctrine of the vascularity of the teeth. Here, at once, I join issue with the doctor, and here *is*, as it were, the foundation stone of dental science. Unless we commence aright here, how shall we expect to erect a superstructure which shall not crumble and fall before the "unerring touch-stone of truth."

On this pivot hangs the fate of our practice, for it is on this the doctor builds his theory of decay, and says if his theory is correct, (and it is put forth with the utmost confidence,) "that the usual notions which prevail on the subject of decay, miscalled caries, must yield to a better and more correct pathology, and consequently to a more scientific and less empirical practice."

On page 44, paragraph 96, we find the following very explicit and frank declaration, (in speaking of the bone of the teeth,) "its vitality is very low, as it possesses neither circulation nor innervation, being kept in a moist state by the serum furnished it by the surface of the pulp."

I hope the doctor will furnish us at some future time with the manner in which this serum enters the bone, or if it keeps it moist merely by contact; and how, when the pulp is removed by obliteration of the dental cavity, is the bone kept in a moist state? I shall look for some more light on this particular subject when *his* "more scientific and less empirical practice" is given to the public.

It becomes us, then, Mr. President, to know whether, in our daily operations, we are handling a living and organized part of the human body, or are we really deceived, notwithstanding the testimony of our senses?

This subject (the vascularity of the teeth) has agitated the medical and particularly the dental profession ever since the days of Hunter. It appears to me rather strange that this eminent philosopher should have taken the view of the subject which he did. I think it the more strange when we find that he practised and recommended the transplanting of teeth. To have been consistent, this practice alone should have decided him in the affirmative of this question, notwithstanding the seeming contradiction of his experiments. Indeed, I have always thought he had his doubts on this subject; for in speaking of the bone of the teeth, he remarks that it is a "mixture of two substances, viz. calcareous earth and an animal substance which we might suppose to be organized and vascular." I believe there are but few practical dentists who have published their views of late on this subject, but teach that the teeth are regularly organized and vascular. I will merely name Fox, Bell, Koecker, Fitch and Harris, who take this view of the subject. Mr. Bell gives some facts which go to prove the existence of absorbing and secreting vessels, and had a case where an abscess was formed within the tooth. A few years since I had a case of *supposed tic douloureux*, which I cured by the removal of a tooth perfectly sound in appearance, and, when split in two, showed a well-formed abscess containing pus.

Let any one engage in the practice of dentistry; let him handle these organs himself, and I am sure if he does not believe in their sensibility, his patients at least will arrive at that conclusion.

Let us, however, examine a few leading facts which go to prove their vascularity. It is contended by Professor Retzius, that the crusta petrosa, or cementum, is the medium through which "nutrient fluids are conveyed to the tooth after the obliteration of the cavitas pulpæ. Even Dr. Goddard, who follows almost exactly in the track of Professor Retzius, admits that the teeth (or ivory so-called) "is kept in a moist state by the serum furnished it by the surface of the pulp." So that at least this portion of the blood (the serum) enters somehow or other the bone of the tooth.

If the teeth are not organized it cannot be by absorption, for then they possess no absorbent vessels. If by capillary attraction, what is the attracting agent? I use this term, capillary attraction, in anticipation of the doctor's theory of endosmotic decay.

How, then, does this portion of the blood separate itself from the crassamentum, and nourish and support the bone of the teeth?

We find other parts of the system, which, in a healthy condition, receive not the red globules or coloring matter of the blood; this is supposed only to be a fibrous gluten and oxydated iron. The particles of coloring matter, being of too coarse a nature, do not, in a healthy condition of the organ, pass into the tubuli of the teeth; neither do we find it the case in the tendons and capsular ligaments. Yet in all of these parts, when laboring under a high degree of inflammation, we find them suffused or colored by the red particles of the blood. It is certainly a beautiful arrangement of a Power, who displays wisdom and skill in all His works, by which the dental organs are made to add beauty and give expression to the face of man. Was it not for this, the teeth would be in color as the muscles of the body, and would not possess that hardness which is necessary in the use for which they were made.

The teeth of young persons, after a severe or protracted attack of odontalgia, on removal, frequently show evident traces of an effusion of blood through the bony structure of the teeth. This shows, that during a high state of inflammation the globules must be somewhat broken up or the tubuli enlarged, for the diameter of the tubuli are estimated at $\frac{1}{4620}$ of an English inch, while the globules of red blood are $\frac{1}{3000}$.

Dr. Harris, of Baltimore, has, I believe, some beautiful specimens of teeth, of this kind, and in page 18, vol. 3, of "American Journal and Library of Dental Science," thus notices a tooth in the possession of Dr. Maynard, of Washington City. He says, "we were shown, a short time since, by Dr. Maynard, of Washington, a molar tooth, the bone of which externally and internally extending more than one-third of the way through the parieties of its roots, was beautifully suffused with red."

"This circumstance," he farther remarks, "conclusively establishes the doctrine, that the investing as well as lining membrane of a tooth contributes to the vitality of tooth bone." My brother, Dr. E. Taylor, of Natchez, Miss., had a tooth, a beautiful specimen of this kind, I believe now in the possession of Dr. Harris, which presents a very similar appearance.

It has often been noticed that the teeth of persons who have died from strangulation are "almost uniformly tinged red."

In the days of the insertion of natural teeth, I had an opportunity to notice this on one occasion, myself. The teeth were so much tinged that they could not be used for dental purposes. In the 3d vol. of the American Journal, page 212, you will find a very interesting letter from the late Dr. Hayden, of Baltimore, to Professor Dungleson, on this subject, which strongly proves the vascularity of dental bone.

We often meet with teeth injured by slight blows or some violence of that kind, which assume a dark appearance in a few days. This cannot be from the action of a chemical agent on the calcareous matter of the teeth, but rather from an effusion of blood and consequent disorganization of the vessels in its bony structure. In this case, often, no fracture of the enamel has taken place. The action of the acids of the mouth have not had time to disorganize the tooth, either by actual contact or by "capillary attraction."

And furthermore, this discoloration is general and pervades the whole tooth, giving, very soon, evident proof of the death of the tooth. It is contended by Dr. Goddard that the white color of the ivory is due to the contents of the tubuli, and that they contain calcareous earth. Now, this discoloration could not be effected, unless a chemical agent could reach the contents of the tubuli. To accomplish this, the enamel must be fractured, or removed, or the doctor's very peculiar process of "capillary attraction" must take place, and that, too, throughout the whole structure of the tooth.

The former does not always take place; grant the latter and you make this "capillary attraction" greater than during a state of decay, and of sufficient force to attract through the enamel itself: every one can make his own inference.

Mr. President, I find it utterly impracticable, in an address of this kind, and in the time I have to devote to this part of the work under consideration, to present and dwell on all the facts which go to disprove the position taken by the doctor on this subject. I, therefore, point out merely some of the main and leading arguments, and leave it to every member of this society to fill up the more minute details, as reason and judgment may dictate.

How can we satisfactorily account for the filling up of the dental cavity of the teeth, as they wear down by mastication or by the process of denudation, if deprived of a circulating fluid and secreting and excreting vessels? I, this day, had a case of this kind, which was a beautiful illustration of the power of the system exerted for her own defence; a deposit of bony matter was thrown over the nerve of almost every tooth, which were thus protected from exposure.

The doctor, in his zeal for ivory, does not even allow the crusta petrosa, a place among the bones of the body, but makes it hold an intermediate rank between ivory and bone, forsooth, and why? "because it lacks the vessels, Haversian canals, and contains less animal matter," and yet in the tables of analysis annexed, he has true bone 33 per cent., ivory 28, and cementum or crusta petrosa 42.18, being 9.18 more animal matter than bone.

But I forbear at present to pursue this subject. The reputation of the doctor, as a medical man and a writer, and consequently the influence his work might be expected to exert, must be my excuse for taking up so much of your time on this particular part of the doctor's book.

The next thing, which I shall notice, is the preparation of the cavity for the plugging of the tooth.

After giving directions in relation to the form of the cavity and the manner of removing the decay, the doctor remarks, that "sometimes the cavity is tender and unfit to receive the plug, and at others, in making the necessary excavations, the operator has reached and exposed the dental pulp," and goes on to say, that "in the former case it is better to defer filling the cavity for a few days, and to place in the cavity a small piece of cotton moistened with a solution of arsenious acid in water." After describing the method of preparing this solution, he goes on to say, "this solution is not very strong, but amply so to fulfil the indication, namely, to destroy the sensibility of the freshly cut and exposed ivory." This sensibility, on page 78, par. 195, he explains as follows: "The tubuli are softened and converted into a state resembling cartilege; the calcareous matter in their calibres is dissolved and replaced by a morbid fluid, which, when the tubuli are compressed by the touch of an instrument, is injected forcibly on to the surface of the pulp and produces the pain spoken of." To enable us to get at the whole of this theory, I will give the remainder of the paragraph, "when the decayed and softened part is entirely cut away, this pain ceases, because the parieties of the healthy tubuli are too rigid to allow this compression to take place. This is commonly attributed to the removal of the sensitive and inflamed bone." "Here," says the doctor, "may be deduced the modus operandi of the solution of arsenic." And here, Mr. President, say I, is "the mountain in a nut shell." The doctor does not tell us the modus operandi of the arsenic, but leaves us to infer it. How then does it destroy the sensibility of the tooth? Does it alter the tubuli and their contents, so that the tubuli are changed from their cartilaginous appearance and resume their natural healthy condition, so that the morbid matter they contain cannot be "injected forcibly on to the pulp?" or is this matter also transformed into healthy calcareous earth? (excuse the term healthy applied to inorganic matter.) I think we can hardly account for it in this way. How then does it take place? Is it by acting upon, and destroying the contents of the tubuli until it reaches the dental pulp, and paralyze or destroy this also? or does it reach the pulp by "capillary attraction," and merely allay the sensibility of the irritated nerve by its soothing power? Is there indeed such a reciprocal attraction between the nerves of our body and this most potent poison, if it acts on the contents of the tubuli? It must, necessarily, leave them in a worse condition than before the application of the arsenic.

For we must recollect that the decayed part is often exceedingly small where this tenderness exists, and that the contents of the tubuli, between the decayed part and the pulp, are yet in a healthy condition. If we say not, how can the plug preserve the tooth, when they are filled with a morbid fluid? This morbid fluid, so exceeding subtile, must be somehow got rid of before filling the tooth, or decay *will go on.* We are generally very careful to remove all moisture from the cavity before filling the tooth. It does appear to me that some more plausible theory than this will have to be established before the matured conclusions of such men as Fox, Bell, and others of scientific research, shall be thrown aside as worthless and "untenable."

I have noticed cases where this remedy had been used, where the dental pulp was destroyed, and periodentitis and alveolar abscess was the consequence. A much better plan, in my opinion, to remove this tenderness, is to take a very sharp excavator, and at one sudden effort, press it to the bottom of the cavity, and by a rotatory, or circular motion, at once remove the softened decay, and paralyse the extreme sensibility; after which you can leisurely cut away the more hardened part of the bone, which has suffered from the disease, and form your cavity without much pain. When done in this way the tooth retains its color after being plugged; which there is no certainty of when the arsenic is used. I have noticed that these tender teeth are generally found in the mouth of young persons, when the bony structure of the tooth has not attained the density and hardness it afterwards acquires. Should the nerve be only slightly covered by healthy bone, a cap can be thrown over it, so that the pressure of the plug will not produce any irritation. When something is absolutely demanded to remove the tenderness spoken of, (which is very rare indeed,) nut gall, either in extract or powder, is far more safe and preferable to arsenic.

Unless we wish utterly to destroy the nerve or pulp, I utterly repudiate the use of arsenic in any shape or form. This leads me to the treatment of the pulp when exposed, as recommended by the doctor. He recommends the use of arsenious acid applied with creosote, and retained by wax and mastic, and remarks that "arsenic used in this way has the advantage of first destroying and then preserving the pulp perfectly, so that no change will afterwards occur in it.

I must acknowledge, Mr. President, that I have not found this to be the case. Most usually a slight suppuration takes place, and this must cease before the tooth can be filled; and even was this not the case, and the pulp merely killed, and then preserved, or, if you please dried up, yet it is just as important to remove this as other dead and foreign matter from the cavity. But wherever the action of the arsenic ceases, there the deadened portion must separate from the living, and this, I might say, invariably takes place at the point of the root.

If the action of the arsenic here ceases, and no inflammation takes place in the periosteum, the tooth, may, for a time, do well; yet, what takes place which occasions the soreness of the tooth in the socket, which the doctor notices, is it not an inflammation of the periosteum? And is it not the irritation and thickening of this membrane which causes the protrusion, as it were, of the tooth? I would here, also, remark, that the more roots and nerves we have to contend with in a tooth, the less likely are we to succeed in our operation. I believe that those who used this remedy most, four or five years since, now scarcely ever use it at all; having found that the most of such teeth prove troublesome, and ultimately have to be removed. They are disposed at once to rid the mouth of such troublesome customers. I have found the cobalt a less painful and equally efficacious remedy, used as the arsenic is recommended, with creosote, &c.; yet I have so little confidence in the utility of such operations that I have never seen proper to make it known publicly as a remedy. How much better for the profession, how much better for the public would it be if no remedy but extraction was relied on for the cure of tooth-ache!

We know, Mr. President, that this nerve-killing business is the very *acme* of a quack's attainments. Indeed, with this remedy, and his royal succedanium, he imagines he can travel the world over, and ride science, rough shod, out of existence. I had hoped that no scientific man could be found who thinks the one worthy of recommendation, or the other deserving of a prolonged and minute description. Unfortunately for the dental profession, much that is pernicious may temporarily relieve the pain and also as a plug appear for a while to be beneficial. So with the mineral paste, (and all pastes, I venture to assert, are mineral, which are used for plugging,) to those ignorant of the action of mercury, with its amalgams on the mouth, might be thought for awhile to be beneficial, yet ultimately it will be found to not answer the desired purpose.

Mr. President, it is not my intention, in this short address, to notice all I consider objectionable in the work under consideration; neither shall I notice particularly what I think useful and scientific. The latter would be a more pleasant task. It is to me far more agreeable to speak in commendation than disapprobation; yet what is thus put forth for the public good, virtually becomes the property of the public, and I consider it the duty of some one to test the principles and theories advanced, and if good, to do all in his power for their promotion. I hope that some one, more capable than myself, will review this work for the benefit of the profession. I hope I shall, therefore, be excused if I only take a passing notice of the remainder of the work.

One remark I wish to make in relation to the use of dilute muriatic acid for the removal of tartar from the teeth. It is true the doctor does not say it is the best manner of getting rid of this troublesome incrustation, yet he says, it "may be thus removed," and is somewhat particular in giving directions how it shall be done. This is also applicable to his notice of "mineral cements," so-called, &c.

Now, what good is to be derived in placing such a work in the hands of students and young practitioners? and it is designed especially for their benefit, for in the preface the doctor says, "it is not presumed that the practical dentist, of age and experience, can desire much from a work like the present," except, indeed in relation to "the microscopic anatomy of the teeth and pathology of decay." To the student, then, this work is especially directed; and he is particularly directed how he may use muriatic acid in the removal of tartar, and also, minutely told how he may prepare his amalgams for the plugging of teeth.

In conclusion, I offer a few remarks on the pathology of decay, which is commended especially to the "practical dentist of age and experience." First, as it respects the action of chemical agents on the enamel and bony structure of the tooth, I have no objection; perhaps no dentist would deny their injurious effect, *even should he* see proper to use an acid in the removal of tartar; at the same time, however, I cannot see how this can invalidate the doctrine of the vascularity of the teeth. Even those who contend for their regular organization, also thus account for their decay. None pretend that they possess a recuperative power and sufficient vitality to resist the action of chemical agents.

Yet many would, perhaps, doubt the theory advanced of decay by capillary attraction. To be more explicit, I shall give this in the doctor's words: "In the case where the decay commences on the surface of the ivory under the enamel, the explanation of the process is slightly different. In this case there is no perceptible fissure or hole in the enamel, but a want of union between its basalt-like columns. A porous condition, indicated by a slight and scarcely perceptible opacity. The ivory at the bottom of this porous spot is bathed in an alkaline serum, furnished by its pulp, whilst the outside of the tooth is constantly exposed either to the contact of acid or acidifying substances. These are the conditions most favorable to the occurrence of endosmosis or transudation ; the alkaline matter exuding, and the acid intruding, simultaneously. The ends of the tubuli, thus bathed in acid, take it up by capillary attraction, and carry it in the direction of the pulp, dissolving the calcareous contents of the tubuli." To keep this process properly in our mind, we must recollect that there is a porous spot on the enamel, and also within the tooth, (of course in the dental cavity,) there is a collection of alkaline serum, which, by some chance or other, fixes itself on the end of the tubuli, the other end of which is exposed by the porous spot in the enamel.

First, then, let me ask what occasions this porous condition of the enamel? Has it not been produced by the action of some corroding substance before this opacity is discernible? If so, the decay is just the same as when some chemical agent has fixed itself in some fissure of the tooth, and thus destroyed the enamel, and then more rapidly the bone of the tooth.

It appears to be a very easy matter for the doctor to establish a circulation of acid, calcareous earth, and serum through the tubuli of the teeth; yet these same tubuli are too small to admit the delicate fibre of a nerve or the passage of the blood. But, above all, they are beautiful and admirable injection pipes, requiring only the slight touch of an instrument to put them in operation. Truly, I think our western and southern physicians should, before they "order," consult the doctor in the purchase of their instruments, and thus avoid "exciting the risibility" of their brethren in the east.

Mr. President, I must confess that, in the perusal of this work, I have been much disappointed, having understood that the doctor's labors run in another field than that of practical dentistry, being demonstrator of anatomy in the University of Pennsylvania, lecturer on anatomy, etc. etc., possessing such opportunities, having already acquired some reputation as a writer, and certainly having ability to do such a work as is needed, justice. I did look forward with considerable pleasure, hoping to see a work supplying that link between medical and dental science which the interest of both, in some measure, demand. A work more pathological than practical, showing the effect of diseased dental organs on the general system. The influence of such disease on the stomach, lungs, the nervous system, and through these on other diseases.

I now dismiss this subject, hoping that my remarks may lead to investigation, by which means alone we may expect to arrive at the truth, and thereby benefit ourselves and the community, who look to us as preservators of organs on which, in some measure, all the rest depend.

